# Island nail flap in the treatment of foot macrodactyly of the first ray in children: report of two cases

**DOI:** 10.1007/s11832-015-0670-z

**Published:** 2015-08-04

**Authors:** Francisco Javier Downey-Carmona, Araceli Lagares, David Farrington-Rueda, José Lirola-Criado

**Affiliations:** Department of Trauma and Orthopedic Surgery, Pediatric Orthopedic Unit, Hospital Virgen del Rocío, Avenida Manuel Siurot s/n, 41013 Seville, Spain; Plastic Surgery, Hospital Virgen del Rocío, Avenida Manuel Siurot s/n, 41013 Seville, Spain

**Keywords:** Macrodactyly, Reconstruction, Island nail flap, Deformity

## Abstract

**Purpose:**

We evaluated the result of a combined single-stage surgery in the treatment of first ray macrodactyly in children.

**Introduction:**

Macrodactyly is a rare congenital abnormality that involves thickening of both the soft tissue and bone of the affected digits. It is more frequent in fingers than toes, where there is less neural involvement. Increased growth is also seen in neurofibromatosis, hemangiomatosis, arteriovenous malformations, congenital lymphedema, and syndromes such as Klippel–Trenaunay–Weber syndrome and Proteus syndrome.

The goal of treatment is to obtain a pain-free, functional foot that can accommodate normal shoes. Treatment of macrodactyly of the first ray generates numerous difficulties since ray resection, which has been recommended for other toes as a means to of shortening and narrowing the foot, cannot be performed. In addition to this, cosmetic results are better if the nail is preserved.

**Methods:**

We retrospectively reviewed our cases of first ray macrodactyly treated by a single-stage multiple-technique procedure.

**Results:**

We obtained satisfactory results, in that same-sized shoes could be worn on by our patients and patients and family were happy with the outcome. However, one of our cases patients lost the nail 10 months postoperatively.

**Conclusions:**

We believe that island-nail transfer in children obtains excellent results.

## Background

Macrodactyly is a rare congenital abnormality that involves thickening of both the soft tissue and bone of the affected digits [1–8]. It is more frequent in fingers than toes, where there is less neural involvement [4]. Nonetheless, the condition is often progressive in both [4]. Etiology and pathogenesis is still unknown [5]. Increased growth is also seen in neurofibromatosis, hemangiomatosis, arteriovenous malformations, congenital lymphedema, and syndromes such as Klippel–Trenaunay–Weber syndrome and Proteus syndrome [1, 2, 7, 9].

Macrodactyly of the foot is usually isolated [2]. Because the number of affected digits and growth and extent of the overgrowth varies, treatment decisions have to be made individually. The goal of treatment is to obtain a pain-free, functional foot that can accommodate normal shoes [4, 10]. Treatment of macrodactyly of the first ray generates numerous difficulties since ray resection, which has been recommended for other toes as a means of shortening and narrowing the foot, cannot be performed [2, 3]. In addition to this, cosmetic results are better if the nail is preserved [3].

The blood supply to the nail unit originates from the proper digital arteries, proximal to the interphalangeal joint, that give off the dorsal digital branch which feeds the nail fold and matrix through the superficial arcade. The nail plate originates from the proximal nail fold and is bordered on either side by the lateral nail folds. The nail matrix is located on the ventral surface of the proximal nail fold. The phalanx is located under the nail without any dermis or subcutaneous tissue between them [10].

We describe our surgical technique in two cases of macrodactyly of the first ray treated by a combination of resection of the distal phalanx, metatarsal shortening, debulking, and island nail transfer in a single-stage operation.

## Materials and methods

We performed a retrospective study of our first two cases of macrodactyly of the first ray treated with the island-flap technique.

### Surgical technique

**Fig. 1:**
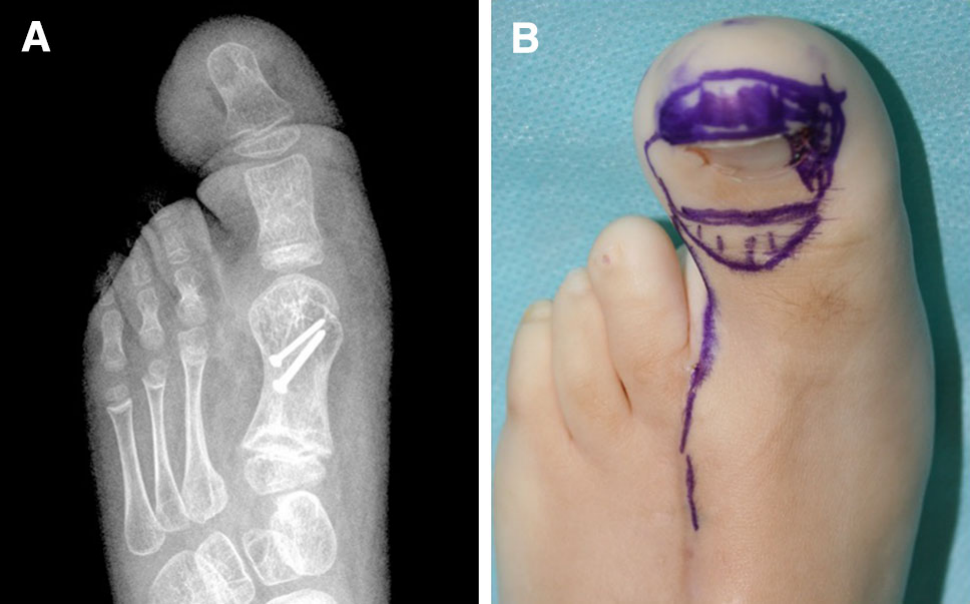
Case 1: Preoperative radiographs (**a**) and planned incisions (**b**). Note the hatched area where the flap was to be transferred

**Fig. 2:**
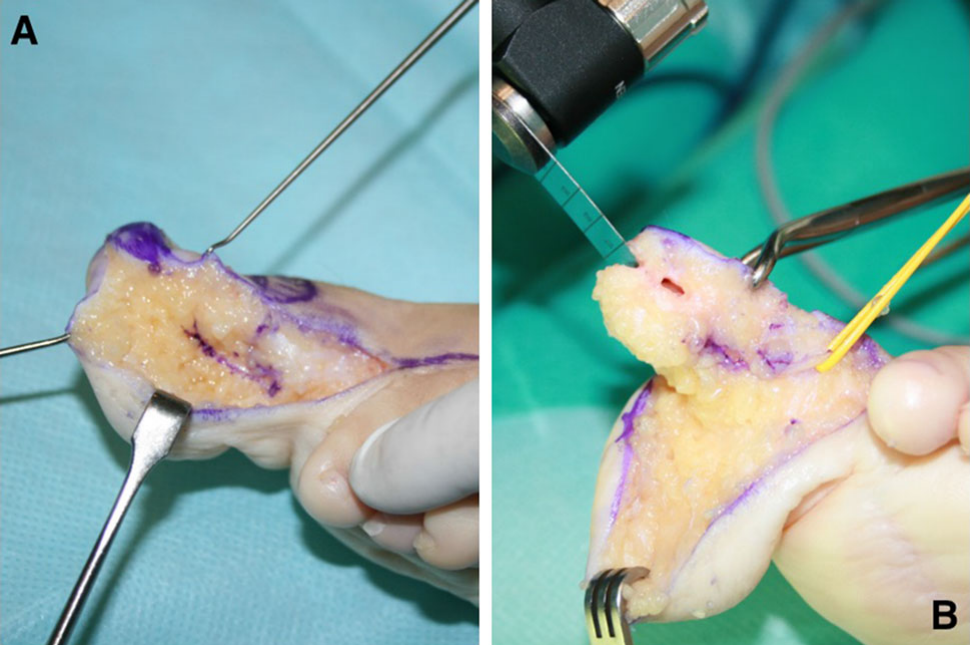
Case 1: Intraoperative images. **a** Dissection of the nail flap. **b** Longitudinal osteotomy of the distal phalanx. Note the vessel loop protecting the neurovascular bundle

Surgery was performed under general anesthesia and tourniquet and fluoroscopic control. The incisions were planned to ensure proper location of the nail bed and proper final toe length (Fig. 1). First, we carefully dissected to preserve the lateral collateral artery. A longitudinal osteotomy of the dorsal aspect of the distal phalanx was performed to obtain a segment of 2–3 mm thickness with the nail complex attached (Fig. 2). Care was taken not to damage the neurovascular bundle while the osteotomy was performed. The flexor and extensor tendons were transferred to the proximal phalanx and the remaining distal phalanx was excised.

After the dorsal side of the proximal phalanx was stripped of periosteum, the island nail graft was mobilized to the proximal phalanx, where the bone–nail complex was fixed with transosseous sutures (Nylon 3/0).

**Fig. 3:**
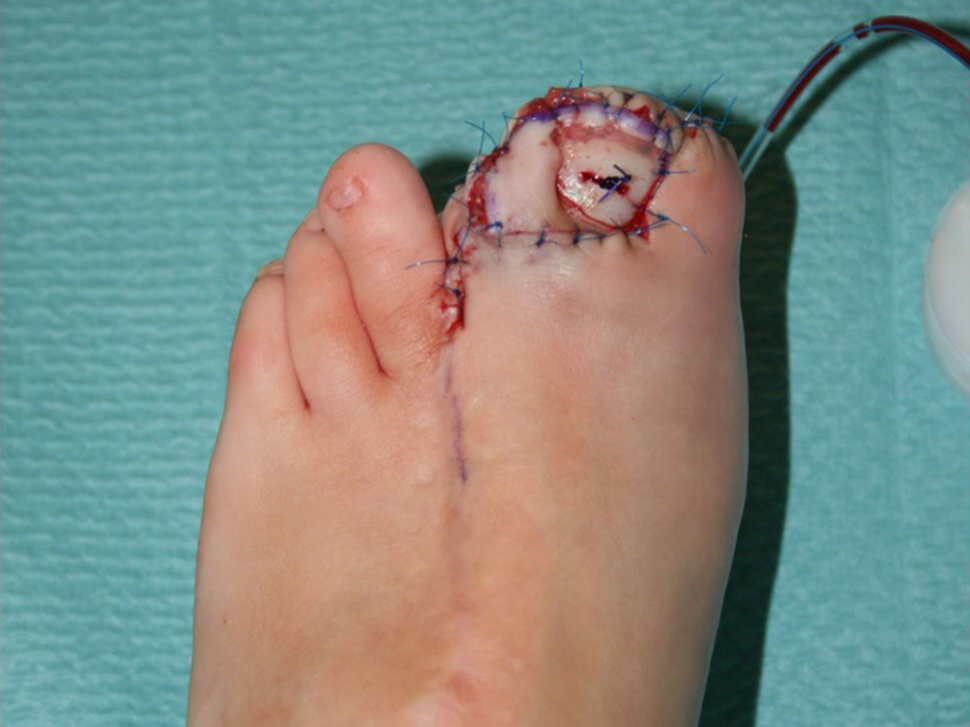
Case 1: Final immediate postoperative result showing adequate perfusion of the toe

After tourniquet release, the skin was closed with interrupted non-absorbable sutures (Prolene 5/0). A bulky dressing and a lower-leg splint were applied over an aspirative drain (Fig. 3).

Postoperatively, vascular status was monitored by simple observation and capillary refill of the tip of the toe. The patient was usually discharged at the third or fourth postoperative day.

### Case 1

A 2-year-old girl was referred to our hospital for evaluation of enlarged first and second toes of her left foot. The Pediatric Orthopedic Deformity Unit and Pediatric Plastic Surgery Unit jointly evaluated her. Clinical and radiographic evaluations confirmed the diagnosis of macrodactyly of the first and second ray. Due to continued, disproportionate growth of the affected rays and difficulty in finding proper footwear, surgery was recommended.

Under general anesthesia and tourniquet control, we performed a complete amputation of the second ray, oblique shortening osteotomy of the first metatarsal, which was fixed with two 1.5- and 2.0-mm screws (Compact hand, Synthes^®^), and debulking. The soft tissues were closed with absorbable sutures (Vicryl 3/0) and the skin was closed with interrupted non-absorbable sutures (Prolene 4/0). An aspirative drain was left and a short leg splint was applied over a well-padded dressing.

Immediate postoperative follow-up was without incident. At 1-month follow-up the short-leg splint was changed for a walking cast and full weight-bearing was authorized.

Due to continued growth of the first ray, a second reconstructive surgery was recommended 1 year after the first. At this surgery, we performed the island nail flap, closing the soft tissues in the same manner as in the prior procedure; a short-leg splint was applied over the dressings.

**Fig. 4:**
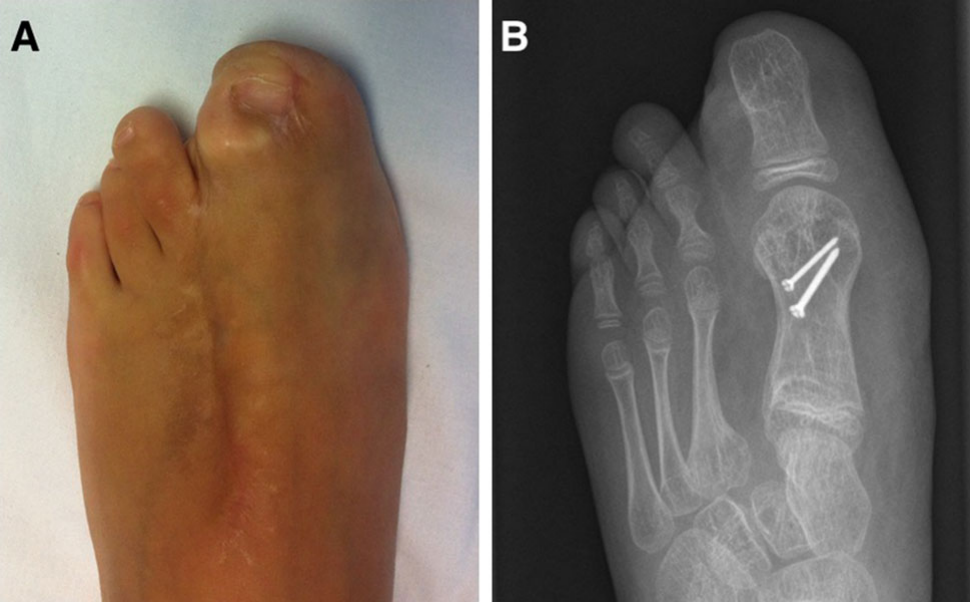
Case 1: Clinical photograph (**a**) and radiograph (**b**) at 15 months follow-up

At 1-month follow-up, the affected foot was longitudinally of the same size as the contralateral one and full weight-bearing was authorized. At 3 months postoperatively, the cosmetic result was excellent: the feet continued to be the same length. However, there was a difference of about 2 cm in width. Nonetheless, she was able to wear shoes of the same size. At present, she is awaiting further debulking surgery but the feet remain the same length (Fig. 4).

### Case 2

A 6-year-old female was evaluated in our clinic. She had been diagnosed with macrodactyly of the first ray with syndactyly of the second and third toes at another center, where a second and third ray amputation was performed at the age of 10 months. Clinically, she presented scars from the previous intervention and presented a lipoma on the anterior surface of the ipsilateral knee. There were no signs of Klippel–Trenaunay syndrome or neurofibromatosis, which led us to diagnose isolated macrodactyly of the foot. The child had trouble with shoe wear and cosmetic issues. Surgical correction was offered.

**Fig. 5:**
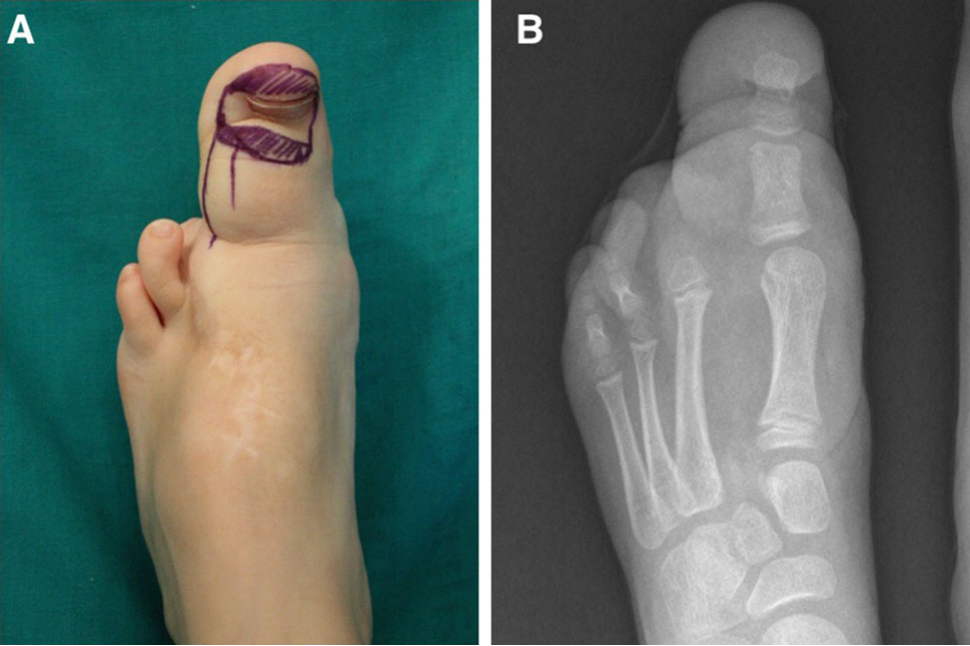
Case 2: Initial clinical photograph (**a**) and radiograph (**b**)

In the operating room, under general anesthesia and tourniquet and fluoroscopic control, the incisions were planned as in the previous case. However, since the lateral collateral artery and vein were carefully dissected but could not be found and the medial vascular pedicle was precarious, the dorsal vein and artery were preserved to ensure proper blood flow to the flap (Fig. 5). The rest of the operation was performed as in the previous case. After tourniquet release, proper blood flow was observed in the flap. The skin was then closed with interrupted non-absorbable sutures (Prolene 5/0). A bulky dressing and a lower-leg splint were applied. Vascular status was monitored by simple observation and capillary refill of the tip of the toe.

**Fig. 6:**
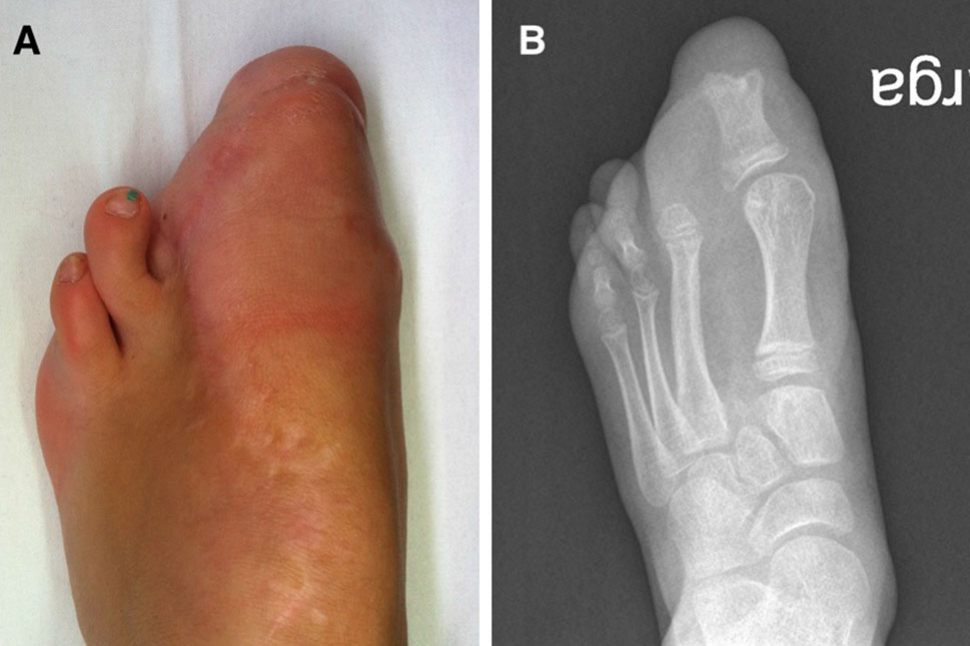
Case 2: Clinical photograph (**a**) and radiograph (**b**) at 1-year follow-up

Initial follow-up was without incident but the nail was lost at 10 months postoperatively. However, the family and child were happy with the result since she wore same-sized shoes. However, as in the previous case she is awaiting debulking of the knee and foot (Fig. 6).

## Discussion

Macrodactyly of the foot is an uncommon congenital malformation in which enlargement of bones and surrounding soft tissues of the foot is observed [1–8]. The deformity is often progressive with less neural involvement than in hand macrodactyly [7]. Barsky [6] made the distinction between two types of macrodactyly: the static type, which does not grow disproportionately, and the progressive type, which enlarges at a faster rate than the rest of the foot. This second type is more frequent in the foot [6, 7].

The main complaints are the impossibility of wearing same-sized shoes and abnormal gait [3]. Therefore, the goal must be to obtain an aesthetically good-looking and “shoeable” foot [4, 6–8]. There is no clear time at which it is best to perform surgery. We think that surgery should be done when shoe wear is difficult or impossible or the foot has become cosmetically unacceptable.

There is general consensus that when the first ray is not affected, the best option is to carry out a complete ray resection [4, 6, 7, 9, 11]. Chang et al. [4] reported that ray resection results in the best functional and cosmetic results when the lesser toes are affected. Since the affected foot is also of increased width, ray resections also address this problem, resulting in a much narrower foot [4, 6].

Since the first ray is so important in foot biomechanics and cosmesis is important (i.e., nail preservation) [8], amputation is out of the question, and reconstructive surgery is therefore indicated [3, 4]. The objective is to reduce the size of the ray and since no one procedure (debulking, epiphyseal arrest, shortening, etc.) can achieve this, many procedures have been developed to be performed simultaneously: debulking, total or partial ray amputation, metatarsal and/or phalangeal shortening, and epiphysiodesis [2–4]. Usually, repeated debulking is needed due to the progressive nature of the deformity [12].

Tsuge [13] described his method of excision of the distal phalanx using a dorsal flap while preserving the nail. The disadvantage is that this method requires 2-stage surgery about 5–6 weeks apart in order to obtain final correction.

Dautel et al. [3] described their technique of island nail transfer in a 9-year-old girl with Proteus syndrome, with excellent results. We used a similar technique but with a variation. The transfer was of the whole nail complex with a portion of bone attached. We think that this should ensure better consolidation and survival of the graft instead of just the periosteum as Dautel et al. describe [3].

Uemura et al. [8] also used an island nail flap to treat a case of macrodactyly of the second toe, with excellent results. As in our case, they transferred the nail complex along with an osseous segment of the distal phalanx.

In our second case, the nail was lost at the 10-month follow-up period. We attribute this to the fact that the child had had previous intervention and blood flow was most likely already compromised.

In our opinion, an island nail transfer, along with additional surgeries, obtains excellent results in first ray macrodactyly. The toe can be shortened as needed since the nail complex, along with a portion of the distal phalanx, can be moved freely as desired. This leads to a higher rate of survival of the graft and, therefore, obtains better cosmetic results.
